# Submarine optical fiber communication provides an unrealized deep-sea observation network

**DOI:** 10.1038/s41598-023-42748-0

**Published:** 2023-09-18

**Authors:** Yujian Guo, Juan M. Marin, Islam Ashry, Abderrahmen Trichili, Michelle-Nicole Havlik, Tien Khee Ng, Carlos M. Duarte, Boon S. Ooi

**Affiliations:** 1 Computer, Electrical and Mathematical Sciences and Engineering, King Abdullah University of Science and Technology, Thuwal, Saudi Arabia; 2 Red Sea Research Center and Computational Biosciences Research Center, King Abdullah University of Science and Technology, Thuwal, Saudi Arabia

**Keywords:** Applied optics, Ocean sciences, Energy harvesting

## Abstract

Oceans are crucial to human survival, providing natural resources and most of the global oxygen supply, and are responsible for a large portion of worldwide economic development. Although it is widely considered a silent world, the sea is filled with natural sounds generated by marine life and geological processes. Man-made underwater sounds, such as active sonars, maritime traffic, and offshore oil and mineral exploration, have significantly affected underwater soundscapes and species. In this work, we report on a joint optical fiber-based communication and sensing technology aiming to reduce noise pollution in the sea while providing connectivity simultaneously with a variety of underwater applications. The designed multifunctional fiber-based system enables two-way data transfer, monitoring marine life and ship movement near the deployed fiber at the sea bottom and sensing temperature. The deployed fiber is equally harnessed to transfer energy that the internet of underwater things (IoUTs) devices can harvest. The reported approach significantly reduces the costs and effects of monitoring marine ecosystems while ensuring data transfer and ocean monitoring applications and providing continuous power for submerged IoUT devices.

## Introduction

The mean depth of the ocean is less than 4 km from the ocean surface, yet our capacity to observe critical properties in the deep-sea lags behind our ability to observe the surface of the Moon or Mars. The workhorse of ocean observing systems (e.g., Copernicus) only penetrates a fraction of the upper ocean. Moreover, oceans cover over 70$\%$ of the Earth’s surface, and the only systematic, global-scale probing into the ocean is the ARGO float system, which only penetrates to 1000 m^1^. Temperature is an essential ocean variable that also informs the rate of change in heat content and heat fluxes from the seafloor^2^, so extending the temperature record below the 1000 m reached by the ARGO float system is essential. A deep-sea observing system should also incorporate information on biological activity, human activity (e.g., mining, trawling, and drilling), and extreme events (e.g., earthquakes and submarine volcanic activity). Ocean soundscapes have been highlighted as an integrative measure of biological, human, and physical processes in the ocean^3^, which could capture the desired metrics synoptically. However, the density and frequency of measurements of essential ocean variables^4^, such as temperature^1^ or soundscapes^3,5^ remain minimal in the deep sea. Therefore, addressing our current gap in observing the deep sea and informing a pending assessment of the environment and ecology of the deep sea and its changes are major goals of the UN Decade of Ocean Science^6^.

The deployment of a conventional sensing system to achieve global coverage of the deep sea would have a considerable cost and face significant technological and operational challenges. Optical-based technology is rapidly rising as a transformative alternative to the sonar-based ocean-related research that has dominated ocean mapping and assessment over the past decade^7,8^, including bathymetry data collection^9^, geological interpretation^10^, navigation^11^, sediment study^12^, and other ocean exploration applications^13,14^. However, sonar activity often has a direct impact on marine life^3^. In contrast, optical-based sensing technology takes advantage of a larger bandwidth, faster transmission speed, lower power consumption, and lower latency than acoustic waves. Fiber-optic sensors have been widely deployed in various ocean monitoring applications^15,16^. For example, distributed fiber-optic sensors enable continuous and real-time monitoring along the entire fiber, whereas the optical fiber network is the backbone of the telecommunication infrastructure over continents. Applying distributed sensing to the telecommunication fiber network can leverage the communication cable for ocean monitoring. In addition to the intrinsic function of optical fibers for data transmission across oceans, optical fiber sensing technology can monitor the ambient environment near the fiber across oceans^17,18^. For instance, optical fiber distributed acoustic sensing (DAS) can achieve seismological data collection^15,19^, geophysical monitoring^20^, earthquake wavefields sensing^21^, and other ocean monitoring data collection applications^16^. Thus, a potential exists to turn the cross-ocean telecommunication fiber network into large underwater distributed sensor networks^18^. Nevertheless, the seafloor already hosts an essential network of data transmissions supporting human operations, as it is crisscrossed by a network of an estimated 1.3 million km of data communication cables^22^. Hence, the SMART subsea cable initiative (Science Monitoring and Reliable Telecommunications) has been proposed to integrate sensors into future undersea telecommunication cables^23^.

The standard single-mode fiber (SMF) and multimode fiber (MMF) are commonly used in sensing and communication applications. Because of the restrictions imposed by nonlinear effects and crosstalk within a fiber core, it is challenging to design a hybrid multiparameter sensing and communication system over a conventional optical fiber^24^. With the emergence of multicore fibers (MCFs) initially designed to provide more capacity for optical networks in the space division multiplexing (SDM) paradigm^25^, multiple signals can be transmitted through different cores of a single fiber. This particular MCF characteristic, which provides minor crosstalk between signals, has initiated the design of multipurpose systems over a single optical fiber rather than installing multiple SMFs or MMFs^26^. The underwater communication network will be progressively replaced by MCFs in response to an expected increase in international data usage by 30–40% from 2020 to 2026, driven by such factors as the growth of fifth-generation mobile data and content shared between data centers distributed worldwide. In October 2021, the NEC Corporation in Japan announced the completion of the world’s first MCF trial, specifically uncoupled four-core submarine fiber cable^27^. The MCF is now expected to further increase the number of parallel optical fiber cores without increasing the submarine cable size and structure, enabling the second generation of submarine SDM systems. However, an interesting research gap remains in accessing a communication MCF to work simultaneously for multiparameter sensing and power-over-fiber (PWoF), a power transmission technology using optical fibers^28^.

**Figure 1:**
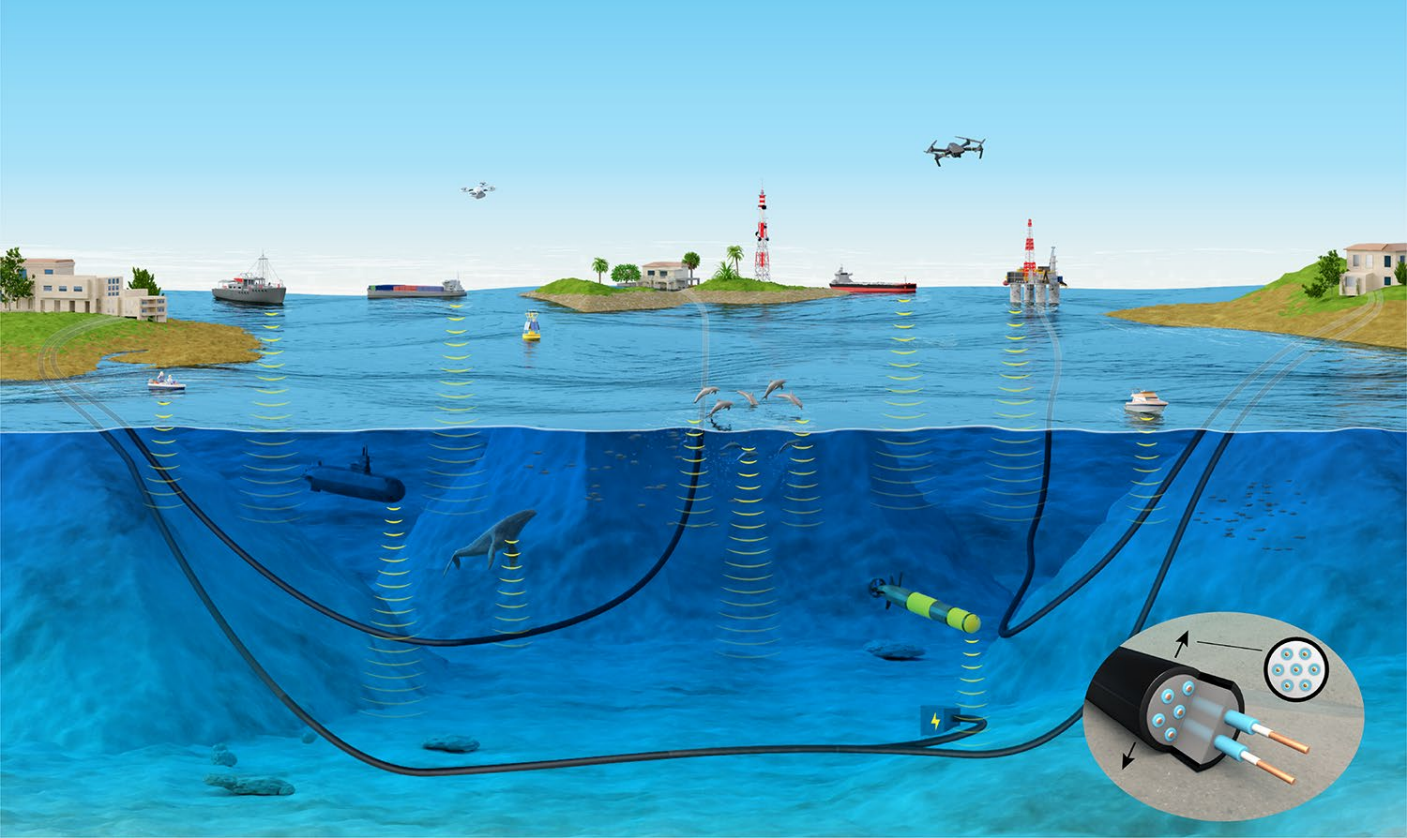
Illustration of a multi-configuration submarine fiber system fulfilling communication, sensing, and PWoF functions.

We provide evidence that the emerging MCF network of cross-ocean telecommunication fiber represents an unrealized ready-to-use observatory of essential variables of the deep sea, without modification, that can typically retrieve temperatures with a high accuracy and soundscape data in the 1 Hz–10 s kHz range. We prove the concept by demonstrating a hybrid submarine fiber-based system that can simultaneously realize multifunctional communication, acoustic sensing, temperature sensing, and PWoF. The integrated system is based on a standard MCF, which can provide unique functionalities that standard SMFs and MMFs cannot offer. In the reported system, a full-duplex (FD) communication is accomplished, a DAS interrogation unit converts the submarine MCF into a distributed vibration sensor, a fiber Bragg grating (FBG) is deployed for temperature sensing, and a photodiode is used in the photovoltaic mode for the PWoF function. While optical communication and sensing have proven tremendous benefits for marine life, PWoF is becoming essential to the underwater environment where the electrical energy supply is constrained. The overall approach of our submarine MCF-based hybrid system is shown in Fig. 1, where an MCF can provide data transmission, transfer energy, and monitor underwater mammal life and the motion of autonomous underwater vehicles (AUVs) and ships. This work would find a myriad of important applications in marine science.

## Methods

**Figure 2:**
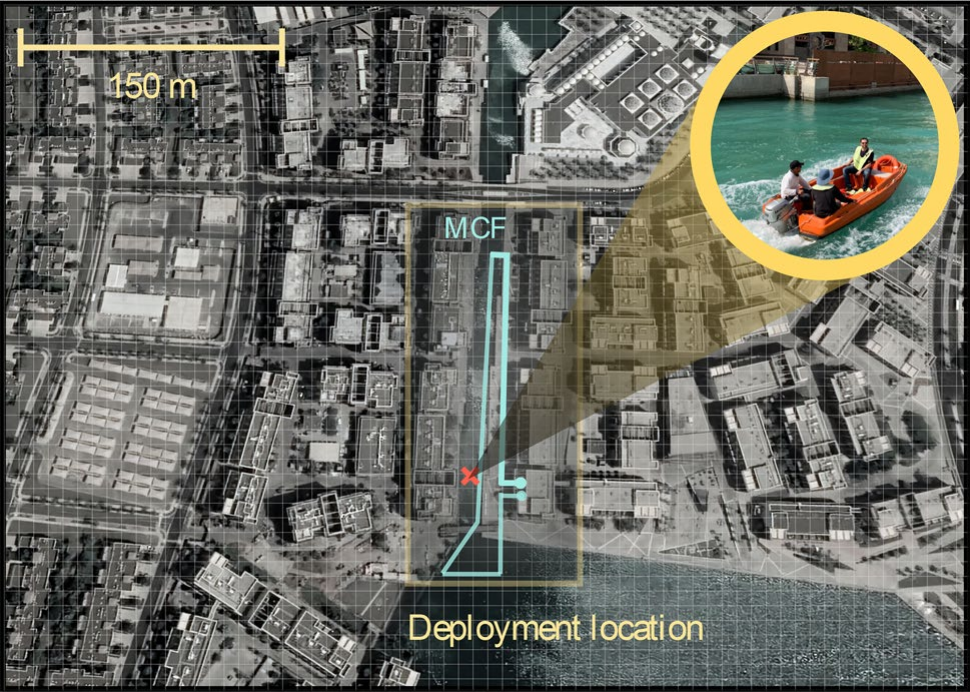
Deployment location of the submarine fiber-optic hybrid communication, sensing, and PWoF system.

**Figure 3:**
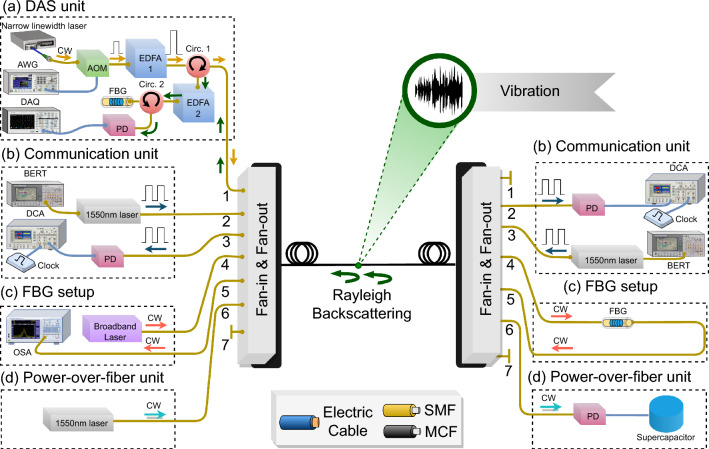
Experimental setup of the submarine MCF-based hybrid communication, sensing, and PWoF system. (**a**) DAS unit, (**b**) communication unit, (**c**) FBG-based temperature sensing setup, and (**d**) PWoF unit.

In this work, data were acquired from a $\sim$1 km long MCF partially placed in a harbor canal at the Red Sea (Fig. 2). While high-speed communication between the two ends of the fiber was applied, the deployment allowed the monitoring of a motorboat movement along the canal, sensing marine life activity near the optical fiber, and transmitting power. In parallel, the temperature sensing from the user end was also demonstrated. Thus, the system’s full functionalities worked simultaneously. The multi-functional system was designed using a seven-core MCF, as illustrated in Fig. 3. One of the seven cores was dedicated to the DAS function, two cores were used to establish FD communication, two cores to the FBG-based temperature sensing, and one core to the PWoF. The MCF fan-in/fan-out injected/extracted light from the individual cores of the MCF.

The DAS technology was designed using the Rayleigh-based phase-sensitive optical time domain reflectometer ($\phi$-OTDR)^29^, which comprised a collection of backscattered Rayleigh traces created by a train of pulses launched from a narrow linewidth 1550 nm laser connected to an acousto-optic modulator (AOM) (Fig. 3). A 5 kHz pulse repetition rate and a 50 ns pulse period were used in the DAS unit, offering a $\sim$5 m spatial resolution. The optical pulses were then amplified by an erbium-doped fiber amplifier (EDFA1) and injected into a core of the MCF throughout a circulator and the fan-in/out multiplexer/demultiplexer (MUX/DEMUX). The backscattered Rayleigh traces from the MCF were circulated to another EDFA (EDFA2), which amplified spontaneous emission (ASE) noise was filtered out using a FBG. The filtered Rayleigh signals were detected by a photodetector (PD) and sampled using a data acquisition device (DAQ).

When there is no disturbance along the MCF, the Rayleigh backscattered signals stay the same in the time domain. But when an acoustic wave hits a point on the MCF, the phase of the backscattered light at that point changes, causing fluctuations in the intensity of the Rayleigh signals only at that point. The position of the acoustic wave on the MCF can be found by using the normalized differential method^30^, in which a normalized differential trace is defined as $\Delta R_i^{norm.}=R_i-R_1/R_1$, $i\in [2,M]$, where $R_1$ represents the first initial Rayleigh trace, $R_i$ denotes the *i*th Rayleigh trace, and *M* is the total number of Rayleigh traces. Also, the frequency spectrum of the acoustic wave can be computed by using the fast Fourier transform (FFT) on the normalized differential traces at that point.

Two other cores of the MCF were used for the FD communication units (Fig. 3). The communication units comprised two 1550 nm lasers directly modulated by a high-speed serial bit error rate tester (BERT). Two balanced PDs were used at the receivers and connected to the digital communication analyzers (DCA) to capture the eye diagram. We chose to work with a data rate of 3.2 Gbps using an on-off keying (OOK) modulation scheme due to the limit of the 3-dB bandwidth of the used PDs equal to 1.6 GHz. The bit error rate (BER) values for each of the two communication links can be obtained by connecting the PD to the BERT, which compares the transmitted pseudo-random bit sequence by the laser to the demodulated bit sequence from the laser beam collected by the PD to compute the error rate.

FBG is a typical fiber-optic sensor with a wide range of applications. Its operation relies on monitoring changes in wavelength, intensity, phase, and polarization of the light transmitted/reflected from the FBG^31^. The Bragg wavelength of an FBG is exploited in temperature and strain-sensing technologies because the spectral FBG reflectivity varies with the temperature and strain changes. In the reported temperature sensing unit, the FBG sensor was connected to two cores from the same side to have a U-turn shape that transmitted a broadband continuous wave (CW) light back to an optical spectrum analyzer (OSA) (Fig. 3). Monitoring the Bragg wavelength shifts on the OSA measurements provided the temperature values at the FBG location. Assuming no strain change along the FBG, the change of the FBG’s Bragg wavelength ($\Delta \lambda _B$) is directly proportional to the temperature change ($\Delta T$), following the formula $\Delta \lambda _B / \lambda _B = \left( \alpha + \zeta \right) \Delta T$,^32^ where $\zeta$ is the thermo-optic coefficient and $\alpha$ is the thermal expansion coefficient of the fiber. We experimentally pre-calibrate the used FBG to find it has a $\sim$9.4 pm/°C temperature sensitivity.

The PWoF unit was included to harvest optical power transferred from one side of the MCF to another in the form of a CW signal (Fig. 3). The PWoF unit contained an indium-gallium-arsenide (InGaAs) photodiode operating in photovoltaic mode to convert the incoming light signal to a photocurrent to charge a 10 F supercapacitor. The PWoF function can potentially be employed to power devices in the internet of underwater things, where changing batteries is costly or not possible. Another merit of the power transfer over fiber is protecting sensitive sensors from electromagnetic interference from power cabling and preventing the risk of electrical hazards. Although this work reports a proof-of-concept demonstration on MCF-based optical power transfer to a unit located at the fiber’s distal end, more units could be placed along the MCF cable and powered through PWoF. The mentioned above MCF fan-in/fan-out can be used to install a unit along the MCF. In particular, an MCF fan-out can be integrated with the MCF at the unit location to extract the PWoF light. Next, a 1$\times$2 fiber-optic coupler can split the optical power delivered by the MCF fan-out into two customized portions, where one of them is delivered to power the unit. The other remaining optical power of the coupler can be coupled again to the MCF through an MCF fan-in. This process can be repeated for installing more units along the MCF.

The number of units that can be powered and placed along the same MCF cable and the maximum distance between the optical energy source and a unit depend on several factors, including the power output of the light source, the attenuation of the fiber, the insertion loss of the fiber-optic coupler and fan-in/out, the efficiency of the photovoltaic power converter (PPC) used, and the power consumption needed for each unit. The typical values of power loss at 1550 nm operational wavelength are 0.25 dB/km MCF attenuation (MCF, YOFC), 1.0 dB fan-in/out insertion loss (fan-in/out, YOFC), 3.4 dB insertion loss for a 50:50 split coupler (TN1550R5A1, Thorlabs, Inc.), and 34.8% efficiency of the PPC^33^. The maximum allowed optical power of the CW light source used for PWoF varies based on the nonlinearity threshold of the fiber core^34^. Considering the design values mentioned above and the power consumption needed for each unit, one can calculate the maximum number of units that can be located along the fiber and their maximum distances from the laser source.

**Figure 4:**
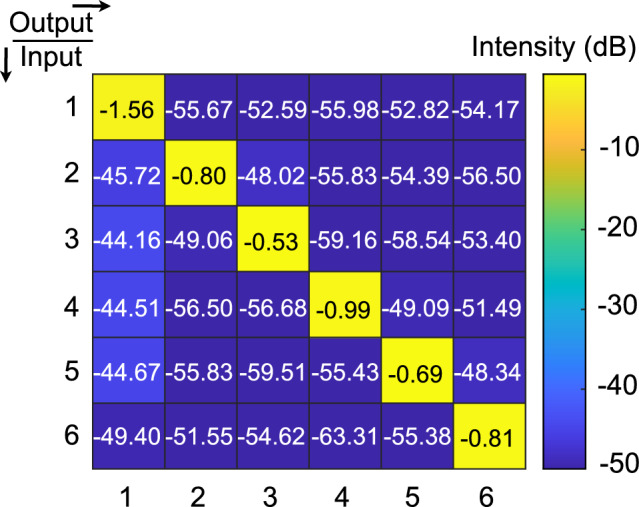
Crosstalk matrix of the MCF used.

The multiple functions of the hybrid fiber-based system were realized through the SDM technology of the MCF. The MCF provides high-capacity optical transmission, but densely-packed cores introduce crosstalk noise that may affect the performance of SDM^35^. At the 1550 nm operational wavelength, the measured crosstalk in our system introduced by the MCF cores is illustrated in Fig. 4. The crosstalk between adjacent cores was measured to be at least -44 dB, which does not significantly affect the performance of the subsystems in this design.

## Results

**Figure 5:**
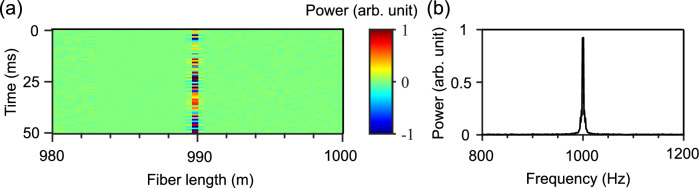
DAS calibration for sensing a predetermined acoustic event. (**a**) Position information and (**b**) power spectrum of a 1000 Hz vibration event produced by the underwater speaker.

*Distributed acoustic sensing* The DAS was running to detect the acoustic event location and frequency to monitor the nearby marine life activities and boat movement near the optical fiber. We initially pre-calibrated the DAS system before the testing by using an underwater speaker (Oceanears, DRS-8), placed $\sim$1 m away from the fiber at a distance of $\sim$990 m from the input port of the fiber. The speaker generated a predetermined sound of a 1000 Hz single-frequency. The location and frequency of different sound sources were identified using the normalized differential method^30^ and the FFT, respectively. As Figs. 5a,b respectively show, the DAS unit perfectly locates the speaker position and determines the sound frequency.

**Figure 6:**
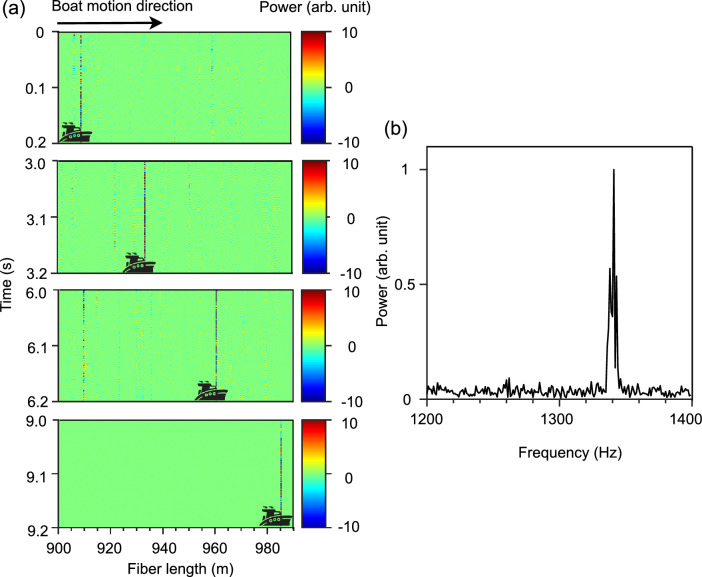
Boat tracking with the MCF-based DAS. The detected (**a**) location and (**b**) frequency of the boat’s sound while it moves in the harbor canal.

The DAS system then detected the sound generated by a boat driven parallel along the KAUST Harbor, mimicking a ship passing near the fiber sensing area. The detected temporal acoustic signal shifted as expected as the boat moved along the fiber on the sea bottom (Fig. 6a), allowing the boat speed, about 8.4 m/s, to be accurately retrieved from the shift in the signal over time. The peak power of the motorboat sound at 1341 Hz (Fig. 6b) agrees well with the characteristic frequency range of small boat sounds^3^. Since the boat was moving parallel to and along the fiber, we here locate the boat in 1-D coordinate (along the fiber). Thus, the location uncertainty is limited by the DAS system’s spatial resolution (5 m), which indicates the capability to separate the Rayleigh backscattering from different positions and thus determines the ability to differentiate adjacent vibration events along the fiber^36^. In other scenarios, such as earthquake epicenter localization using fiber-optic DAS^37^, triangulation techniques are typically used to locate the vibration event in 2-D coordinates (latitude and longitude), and the error is determined with respect to the GPS coordinates.

**Figure 7:**
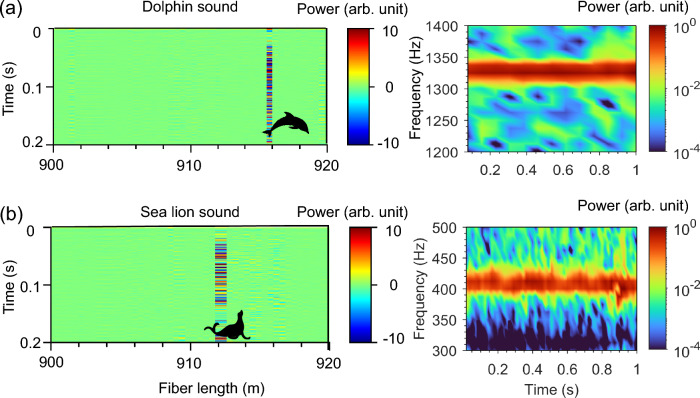
Detecting the sounds of marine mammals using the MCF-based DAS. The detected temporal and spectrogram sound produced by an underwater speaker while playing (**a**) dolphin and (**b**) sea lion sounds.

To emulate the expected marine life in the ocean, dolphin and sea lion sounds were played back by the underwater speaker (Oceanears, DRS-8), located $\sim$1 m away from the MCF at a distance of 910–920 m from the front end of the fiber. As recorded by the MCF-based DAS, the temporal and spectrogram information of the sounds associated with the dolphin and sea lion are shown in Figs. 7a,b, respectively. The dolphin sound has higher frequency components with peak power at $\sim$1329 Hz, compared with the low-frequency sound of the sea lion at a maximum power of $\sim$406 Hz. Such temporal and spectrogram data recorded by the DAS system could be combined with machine learning (ML)^38^ to distinguish the sounds of various marine mammals.

**Figure 8:**
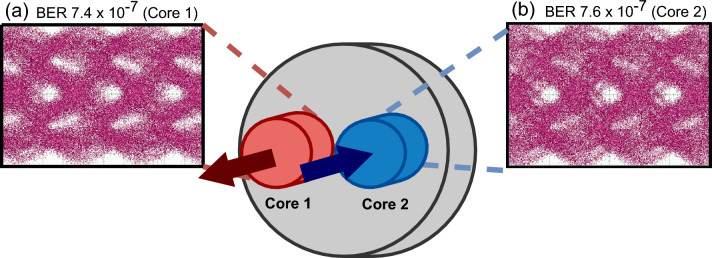
Performance of the FD MCF-based communication system. Eye diagrams of the OOK signals in (**a**) Core 1 and (**b**) Core 2 of the MCF.

*Full-duplex communication* We measured the eye diagrams of the 3.2 Gbps OOK-modulated signals for the FD communication system over two fiber cores, labeled as ‘Core 1’ and ‘Core 2’, (Figs. 8a and 8b), when all other systems were operating in parallel. The open eyes in Fig. 8 indicate good received signal quality on both fiber cores. The high-quality FD communication was achieved with a BER retaining around $7.4\times 10^{-7}$ and $7.6\times 10^{-7}$ for ‘Core 1’ and ‘Core 2’, respectively, which are well below the forward error correction limit of $3.8\times 10^{-3}$.

**Figure 9:**
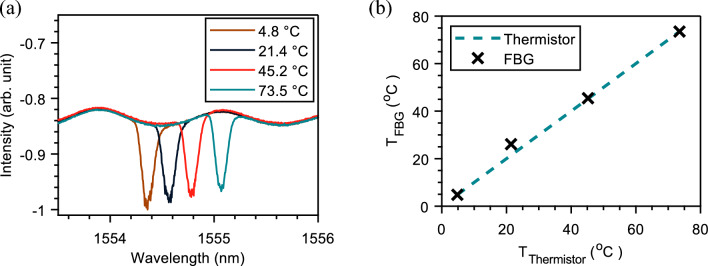
FBG-based temperature sensing. (**a**) Transmitted spectrum through the FBG sensor at different temperature values. (**b**) Comparison between the water temperature values measured by the FBG and thermometer.

*FBG-based temperature sensing* In the experimental design of Fig. 3, the FBG sensor was placed at the end-user side to monitor the environmental temperature. To mimic the possible change in environmental temperature, we placed the FBG in a water bath where its temperature was controllable. Fig. 9a depicts the spectral shift of the FBG transmissivity with the water bath’s temperature changes, as measured by using a commercial thermometer. The measured Bragg wavelengths can estimate the temperature values at the end-user using the precalibrated Bragg wavelength-temperature relationship of the FBG. For the measured Bragg wavelengths, the temperature values calculated using the FBG match those measured by the commercial thermometer well (Fig. 9b). The proof-of-concept experiment demonstrated in this work can be improved by deploying FBG-based sensors with spectrally separated Bragg wavelengths. If that is the case, the FBGs can be installed at distinct interconnected points between MCFs underwater using the MCF fan-in/fan-out components to achieve a quasi-distributed temperature/strain sensing, conditioning compensating for the cross-sensitivity of FBG to temperature and strain^39^.

**Figure 10:**
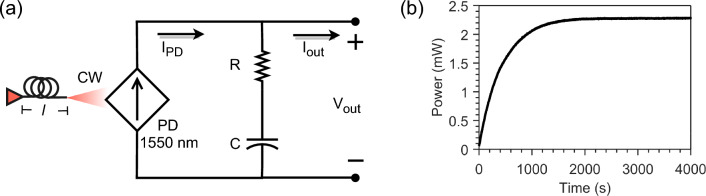
Power transmission over the MCF. (**a**) Equivalent charging circuit of the PWoF unit. (**b**) The supercapacitor power charging curve recorded during the power transmission.

*Power-over-fiber* The PWoF feature of the designed hybrid sensor was tested similarly in parallel with deploying the DAS, FD communication, and FBG-based temperature sensing. The InGaAs PD connected to the end of one fiber core converted a 12 mW received CW optical power into an electrical current to charge the supercapacitor connected in series with the PD. The equivalent charging circuit of the supercapacitor is shown in Fig. 10a. $I_{\text {PD}}$ is the photocurrent from the InGaAs PD working in a photovoltaic (PV) mode and is proportional to the incident light power from the far end of the fiber core. *R* represents the equivalent resistor in the circuit from the supercapacitor and electrical cable, *C* denotes the capacitance of the supercapacitor serving as an energy storage/buffer unit, and $I_{out}$ is the current that feeds the supercapacitor. Fig. 10b presents the supercapacitor power charging curve recorded during the power transfer.

## Discussion

We present a novel design for a hybrid communication, DAS, FBG-based temperature sensing, and PWoF system over an MCF that can form the basis of an emerging global ocean sensing network without requiring significant modification or deployment of additional sensors along the network. As a proof-of-concept demonstration, we report a successful FD communication at 3.2 Gbps while monitoring soundscapes that were able to retrieve components of ocean acoustics, such as marine mammal sounds and boat motion, and sensing environmental temperature. Moreover, in parallel with the applications above, we achieve PWoF using a CW light and charging circuit.

Regarding any potential concern about the impact of the additional functions presented in this work on the communication performance, two main options exist for integrating the reported sensing and PWoF schemes with communication. The first option is to tap into the MCF dark cores, unused cores that do not carry any communication signals and function as a backup for the already used cores. By using dark cores, we can avoid crosstalk with the communication signals in the other cores. The second option can be adopted when the communication signals occupy all the MCF cores. Under the latter scenario, we can use the mature wavelength division multiplexing (WDM) technology to add/drop sensing and PWoF signals on/off the MCF-based communication system. WDM consists of multiplexing multiple optical signals onto a single optical fiber core using different wavelengths^40^. By using WDM, we can separate the sensing and PWoF signals from the communication signals and route them to other devices. Therefore, our reported MCF-based multi-functional technology should not affect the primary purpose of the MCF, which is the communication. On the contrary, it can enhance the functionality and efficiency of the fiber by enabling additional applications.

Oceans contain millions of kilometers of fibers to satisfy the global connectivity demand; thus, the presented concept using the MCF dark cores demonstrates that underwater optical fibers can become sensor networks across oceans without modification to the planned MCF deployment, bridging the gap in deep-sea observation at no additional cost to society. Therefore, the reported design will pave the way for communication and ocean-sensing convergence over the same fiber networks. Due to the excellent capability of simultaneous data communication, distributed acoustic sensing, and temperature monitoring, the proposed MCF-based hybrid sensing approach has excellent potential for long-range real-time ocean monitoring. The system can also be useful in coastline monitoring applications for security surveillance and illegal boating and fishing activity. In addition, energy transfer in the form of light waves has many benefits, including providing continuous power to devices in a harsh underwater environment and eliminating the need to run a conductive copper wire into a high-ground potential rise zone. The transferred power can be stored and self-consumed by other systems.

Breaking through the lack of capacity to deliver globally-sustained deep-sea observations is a crucial goal as we advance along the UN Decade for Ocean Science^41^. The submarine optical communication system presents an unrealized potential to achieve this goal, particularly for MCFs gradually replacing SMF and MMF cables. We should tap into dark cores in an MCF or use WDM to deliver sensing signals and power to achieve this potential. For example, DAS, distributed temperature sensing, and distributed strain sensing require interrogation units placed onshore to launch sensing signals in the dark cores without modification in the existing MCF-based communication network. Signal processing of optical fiber distributed sensing systems is typically performed in real time with moderate computational resources within the interrogation units. Similarly, for PWoF, a light source placed onshore can power a submerged circuit through a dark core. Besides optical fiber distributed sensing and PWoF, other optical and electronic point sensors could also be integrated over an MCF. For example, salinity, pH, chemical, oxygen, and chlorophyll point sensors can be used for deep-sea data collection, and the generated data can be transferred onshore via an MCF-based communication network.

## Data Availability

The data that support the findings of this study are available from the corresponding author upon a reasonable request.
